# CO-CREATING A GUIDELINE FOR MANAGING CHALLENGING BEHAVIOR OF MIGRANTS WITH DEMENTIA AT HOME

**DOI:** 10.1093/geroni/igac059.1426

**Published:** 2022-12-20

**Authors:** Leontine Groen- van de Ven, Serena van der Kaaij, Geraldine Visser, Judith Huis in het Veld, Robbert Gobbens, Simone de Bruin

**Affiliations:** Windesheim University of Applied Sciences, Zwolle, Overijssel, Netherlands; InHolland University of Applied Sciences, Amsterdam, Noord-Holland, Netherlands; Windesheim University of Applied Sciences, Zwolle, Overijssel, Netherlands; InHolland University of Applied Sciences, Amsterdam, Noord-Holland, Netherlands; InHolland University of Applied Sciences, Amsterdam, Noord-Holland, Netherlands; Windesheim University of Applied Sciences, Zwolle, Overijssel, Netherlands

## Abstract

Challenging behaviour is common in people with dementia at home. Nurses guide people with dementia and their informal caregivers managing challenging behaviour. However, common nursing guidelines for challenging behaviour in home care are unsuitable to the situation of migrants with dementia. We started a co-creation project with home care nurses, dementia case managers, informal caregivers of migrant background and student nurses as co-researchers that aims to develop an appropriate guideline. Semi-structured interviews at the start explored the current experiences with challenging behaviour. Interview results informed several working sessions where we discussed the results and used creative working methods such as role play to develop a first prototype of the guideline. The prototype was implemented in the daily practice of six home care teams and followed up during reflection meetings with our co-researchers. Our guideline puts special attention to getting familiar with each other before taking steps in managing challenging behaviour.

